# Investigation of MicroRNA Expression Levels in Peripheral Blood of Turkish Males with Cocaine Use Disorder

**DOI:** 10.3390/epigenomes10030047

**Published:** 2026-07-08

**Authors:** Süheyla Ayfer Arslan, Selda Mercan, Günay Çetin, Hasan Mırsal

**Affiliations:** 1 Department of Forensic Medicine, Faculty of Medicine, Istanbul Aydın University, Istanbul 34295, Türkiye; 2 Institute of Forensic Sciences and Legal Medicine, Istanbul University-Cerrahpaşa, Istanbul 34500, Türkiye; mercans@iuc.edu.tr; 3 Department of Psychiatry, Balikli Greek Hospital, Istanbul 34020, Türkiye; cetingunay@hotmail.com (G.Ç.); hmirsal@superonline.com (H.M.)

**Keywords:** cocaine, cocaine dependence, microRNA, peripheral blood, real-time polymerase chain reaction

## Abstract

**Background:** It is observed that there are a limited number of scientific studies investigating the effect of cocaine use disorder on microRNA (miRNA) levels in human peripheral blood. This study aimed to identify candidate miRNAs that may play a role in the regulation of cocaine addiction by detecting changes in the expression of some miRNAs (miR-9-5p, miR-26b-5p, miR-132-3p, and miR-134-5p) in the peripheral whole blood of cocaine addicts. **Methods:** Peripheral blood samples were collected from 12 Turkish male individuals with cocaine abuse, 11 Turkish male individuals undergoing treatment for cocaine abuse, and 16 healthy Turkish male individuals without any substance abuse. The change in the expression of microRNAs was determined by quantitative real-time polymerase chain reaction (RT-qPCR). In statistical analyses, ΔCt values were analyzed for the expression of miRNAs. Receiver operating characteristic (ROC) analysis was used to assess the diagnostic adequacy of peripheral blood miRNAs. **Results:** miR-132-3p and miR-134-5p were downregulated in the addict group compared to the control group (*p* < 0.05). The areas under the curves (AUCs) of the ROC curve of miR-132-3p and miR-134-5p were significant at 0.778 and 0.744, respectively. **Conclusions:** This study suggests that miR-132-3p and miR-134-5p may have function as therapeutic markers in the treatment of cocaine use disorder.

## 1. Introduction

Substance abuse directly affects millions of people every year, leading to a decline in quality of life, impaired health, and death. Furthermore, substance abuse poses significant economic and public safety challenges to society [1]. Challenges remain in elucidating the mechanisms of substance dependence, as well as in its prevention and treatment [2]. It is known that long-term use of addictive substances modulates gene expression profiles and leads to pathological neuroadaptation in brain regions associated with the reward system and memory [3].

A significant portion of research on substance abuse has focused on miRNAs [4]. miRNAs are short, single-stranded ncRNA molecules ranging in length from 15 to 22 nucleotides. They can regulate gene expression at various levels; they typically bind to the 3′UTR region of the target mRNA and negatively affect protein expression through mRNA degradation or translational repression. However, they can also interact with other regions such as the 5′UTR, coding sequence, and gene promoters. miRNAs with different cellular localizations and binding sites may exhibit distinct functions at the post-transcriptional level by activating translation under specific cellular conditions. The suppression or activation of translation in dynamic miRNA–mRNA interactions depends on the target region, subcellular localization, and cellular responses [5,6]. A change in the expression level of a single miRNA can affect more than 100 genes [7]. Genes encoding miRNAs or miRNA clusters may be located in introns, intergenic regions, and exons and, when co-expressed under the control of common regulatory sequences, can form tissue-specific expression patterns [8].

A large number of miRNAs are expressed in the brain [9]. Previous studies have shown that miRNA-mediated regulation can vary depending on time, brain region, and cellular context during cocaine exposure and withdrawal [10].

Following self-administration of cocaine, it was found that striatal miR-212 regulates cocaine intake via CREB signaling, and that the increase in miR-132 and miR-212 expression persists during cocaine withdrawal [11,12]. In addition, it has been reported that hippocampal miR-134 and miR-26b levels fluctuate during the acquisition and extinction phases of cocaine-conditioned place preference, while acute cocaine exposure reduces miR-9-5p expression in a dopaminergic cell model [13,14].

Following prolonged self-administration of cocaine, changes in miRNA expression in the infralimbic and prelimbic prefrontal cortex and the nucleus accumbens regions of rats were evaluated during acute withdrawal and a four-week abstinence period. It has been reported that the miRNAs showing altered expression may be associated with neurotrophin, MAPK, and glutamatergic signaling pathways [15]. miRNA and mRNA sequencing was performed in the prefrontal cortex of mice that developed a cocaine-conditioned place preference. It was demonstrated that cocaine alters the functioning of miRNA–mRNA networks associated with GABAergic and glutamatergic synapses, calcium signaling, circadian regulation, and axon guidance [16]. The effect of the Translin/Trax complex—which plays a role in miRNA degradation in mice—on the cocaine response was investigated. It was found that this mechanism could alter cocaine-induced locomotor sensitization by modulating specific miRNAs and RGS8 expression in the nucleus accumbens. miRNA, mRNA, and protein data from postmortem ventral striatum tissue of individuals with cocaine use disorder (CUD) were analyzed collectively, and changes in metabolic pathways, oxidative phosphorylation, glutamatergic signaling, and astrocyte–neuron communication were examined [17].

These data demonstrate that cocaine-associated miRNA regulation involves not only changes in miRNA expression but also miRNA–mRNA interactions and miRNA degradation mechanisms. These processes have been reported to be linked to synaptic transmission, cellular signaling, energy metabolism, and glia–neuron communication [18]. However, most current studies are based on animal models, postmortem brain tissue, and bioinformatic analyses.

Most epigenetic modifications are tissue-specific, and the limitations of studying brain tissue to identify epigenetic abnormalities in addiction restrict our ability to elucidate brain changes in living subjects [19]. To date, most research in the field of cocaine addiction has generally been conducted on laboratory animals, and in recent years, human postmortem brain tissue has been used in particular [12,13,14,20]. Currently, it is not possible to conduct similar studies directly on live brain tissue from cocaine-dependent individuals [21]. To accurately and precisely detect substance abuse in individuals, objective blood biomarkers that are sensitive, reliable, and easy to detect need to be developed [22,23]. Therefore, since access to brain tissue is not easy, it is important to conduct blood-based expression profiling studies on substance dependence to develop potential biomarkers for addiction [19]. In particular, studies conducted over the past decade have demonstrated that miRNAs are stable in serum/plasma and may be suitable as blood-based biomarkers for both cancerous and non-cancerous diseases [24,25]. Given the role of miRNAs in addiction associated with substance misuse, to date, there have been very few studies focusing on the expression profiles of serum and/or plasma miRNAs in heroin and/or methamphetamine use disorders [18]. In particular, there is only one study examining the effect of CUD on miRNA levels in peripheral blood [26]. Furthermore, numerous studies have reported that cocaine exposure in rodents alters (increases or decreases) miRNA expression levels in many brain regions [27,28]. For example, some of these miRNAs include miR-9, miR-19, miR-26, miR-34, miR-124, miR-132, miR-134, miR-135, miR-181, miR-183, miR-212, miR-375, miR-449, and Let-7d. In general, it has been reported that these miRNAs may serve as potential biomarkers for cocaine-induced CUD [26,28].

To the best of our knowledge, no study has investigated the effect of CUD on peripheral miRNA levels in male individuals. Therefore, to avoid any confounding effects related to the gender parameter, only male individuals were included in this study. This study represents the first investigation of peripheral miRNA profiles in male individuals with CUD. The aim of this study was to identify candidate miRNAs that may play a role in the pathogenesis of cocaine addiction by detecting changes in the expression of miR-9-5p, miR-26b-5p, miR-132-3p, and miR-134-5p—which have not been previously investigated—and, in particular, to elucidate the relationship between cocaine addiction and peripheral blood miRNA levels. Additionally, this study aimed to investigate the effects of treatment tools and methods recommended for CUD on miRNA levels and to detect changes in miRNA levels before and after treatment. Therefore, the findings from our study suggest that miRNAs may serve as biomarkers for CUD and potential candidates for future targeted therapies. However, it is important to conduct more comprehensive, independent, and long-term studies for their clinical application.

## 2. Results

### 2.1. Participant Characteristics

Sociodemographic and clinical characteristics of the sample are shown in Table 1 below. In the study, a significant difference was found between the addict group and the control group in terms of age variable (*p* < 0.001), also a significant difference was found between the treatment group and the control group (*p* = 0.004), no significant difference was found between the addict group and the treatment group (*p* > 0.05). There was no significant difference between the groups in terms of height (F = 0.439; *p* > 0.05). While a significant difference was found between the addict group and the control group in terms of weight (*p* = 0.008), no significant difference was found between the treatment group and the control group, and also between the addict group and the treatment group (*p* > 0.05). In terms of BMI, there was a significant difference between the addict group and the control group (*p* = 0.006), there was a significant difference between the treatment group and the control group (*p* = 0.050), and there was no significant difference between the addict group and the treatment group (*p* > 0.05). The substance abuse histories of the individuals with cocaine abuse are presented in Table 1. Among the 12 cocaine-dependent patients, 4 had a history of cocaine and cigarette use, 5 had a history of cocaine, cigarette, and alcohol use, and 3 had a history of cocaine, cigarette, alcohol, and cannabis use. Of the 11 individuals who received CUD treatment, 1 used only cocaine; 2 used cocaine and cigarettes; 5 used cocaine, cigarettes, and alcohol; 1 used cocaine, cigarettes, and cannabis; and 2 used cocaine, cigarettes, alcohol, and cannabis. In total, seven (63.6%) patients completed the first detoxification treatment, and the majority of patients (90.9%) received detoxification treatment.

### 2.2. MicroRNA Expression Profiles in Peripheral Blood of Individuals with CUD and Individuals on CUD Treatment

**Figure 1:**
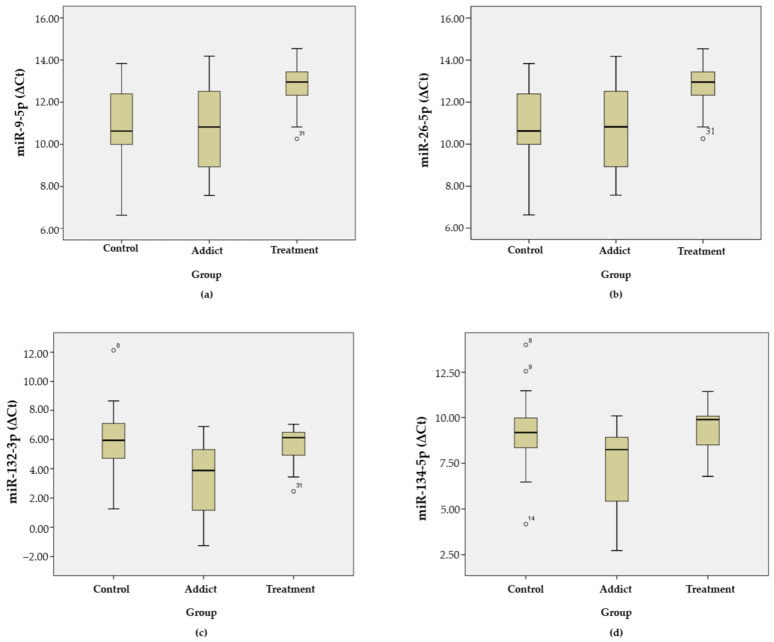
Box plots of ΔCt values for target miRNAs in the control, addict, and treatment groups: (**a**) [miR-9-5p]; (**b**) [miR-26-5p]; (**c**) [miR-132-3p]; (**d**) [miR-134-5p]. The box represents the interquartile range, with the median indicated by the center line; whiskers show the range, and circles indicate outliers. The numbers adjacent to the circles denote the corresponding participant and do not represent measurement values.

MicroRNA expression results were analyzed in addict, treatment and control groups. The relative blood levels of miRNAs in the groups and the comparison results of ΔCt values between the groups are shown in Table 2. Peripheral blood miR-9-5p levels did not show a statistically significant difference in the addict group compared to the control group (*p* > 0.05), whereas they were upregulated in the treatment group (*p* = 0.019). miR-9-5p level was found to be upregulated in the treatment group compared to the addict group (*p* = 0.012). Peripheral blood miR-26b-5p levels were not statistically significantly different in the addict group compared to the control group (*p* > 0.05), whereas they were upregulated in the treatment group (*p* = 0.024). miR-26b-5p level was found to be upregulated in the treatment group compared to the addict group (*p* = 0.009). Peripheral blood miR-132-3p levels were not statistically significantly different in the treatment group compared to the control group (*p* > 0.05), whereas they were downregulated in the addict group (*p* = 0.003). miR-132-3p level was found to be upregulated in the treatment group compared to the addict group (*p* = 0.026). Peripheral blood miR-134-5p levels were not statistically significantly different in the treatment group compared to the control group (*p* > 0.05), whereas they were downregulated in the addict group (*p* = 0.020). miR-134-5p level was found to be upregulated in the treatment group compared to the addict group (*p* = 0.023). The ΔCt distributions of the target miRNAs across the control, addict, and treatment groups are shown as box plots in Figure 1a–d.

### 2.3. Correlation Analysis of Clinical Characteristics with miRNA Expression

In this study, the correlation of clinical characteristics of addict, treatment and control group individuals with ΔCt values of miR-9-5p, miR-26b-5p, miR-132-3p and miR-134-5p, which were selected as candidate miRNAs, were examined. The relationships between age and the ΔCt values of these four candidate miRNAs were examined in the addict, treatment, and control groups, and no statistically significant relationship was found (*p* > 0.05). The relationships between age and the ΔCt values of these four candidate miRNAs were tested in the addict, treatment, and control groups, and no significant effect was observed (*p* > 0.05). When the relationships between BMI and the ΔCt values of candidate miRNAs were tested and analyzed, a weak negative correlation was observed between BMI and miR-132-3p; however, this correlation was not statistically significant (r = −0.132; *p* = 0.422). No statistically significant relationship was found between the ΔCt values of the other candidate miRNAs and BMI either. The relationship between ΔCt values of candidate miRNAs and certain clinic-pathological factors in individuals included in addiction and treatment groups was analyzed using Spearman correlation. No statistically significant correlation was found between the expression of candidate miRNAs in addict and treatment groups and the duration of use, daily dose, type of cocaine used, frequency of use, or characteristics of last use (*p* > 0.05).

### 2.4. Age- and BMI-Adjusted miRNA Expression Analysis

To control for the potential confounding effects of age and BMI differences between the addict, treatment, and control groups on miRNA ΔCt values, ANCOVA analyses were performed for each miRNA, with age and BMI included in the model as covariates. For miR-9-5p, miR-26b-5p, miR-132-3p, and miR-134-5p, where group × age and group × BMI interactions were investigated, no statistically significant effects were found, and the assumption of homogeneity of regression slopes was met. In the resulting models, the fact that the Levene tests were also not statistically significant indicated that the assumption of homogeneity of error variances was met.

When the potential confounding effects of age and BMI were controlled for together, the overall group effect on miR-9-5p ΔCt values was found to be statistically significant (F(2,34) = 5.156; *p* = 0.011; partial η^2^ = 0.233). In contrast, the independent effects of age (F(1,34) = 1.955; *p* = 0.171; partial η^2^ = 0.054) and BMI (F(1,34) = 0.157; *p* = 0.694; partial η^2^ = 0.005) were not found to be significant. The age- and BMI-adjusted means were calculated as 10.573, 10.955, and 13.079 for the control, addict, and treatment groups, respectively. In Bonferroni-corrected pairwise comparisons, the ΔCt values of the treatment group were found to be significantly higher than those of the control group (*p* = 0.029) and the addict group (*p* = 0.028), while no significant difference was detected between the control and addict groups (*p* = 1.000).

When age and BMI were controlled for together for miR-26b-5p, the overall group effect was found to be statistically significant (F(2,34) = 5.516; *p* = 0.008; partial η^2^ = 0.245). The independent effects of age (F(1,34) = 2.444; *p* = 0.127; partial η^2^ = 0.067) and BMI (F(1,34) = 0.560; *p* = 0.460; partial η^2^ = 0.016) were not found to be significant. The adjusted means were calculated as 0.106, 0.388, and 3.527 for the control, addict, and treatment groups, respectively. The ΔCt values of the treatment group were found to be significantly higher than those of the control group (*p* = 0.030) and the addict group (*p* = 0.017); no significant difference was detected between the control and addict groups (*p* = 1.000).

When age and BMI were controlled for together for miR-132-3p, the overall group effect was found to be statistically significant (F(2,34) = 3.880; *p* = 0.030; partial η^2^ = 0.186). No significant independent effects of age (F(1,34) = 0.028; *p* = 0.869; partial η^2^ = 0.001) or BMI (F(1,34) = 0.018; *p* = 0.895; partial η^2^ = 0.001) were detected. The adjusted means for the control, addict and treatment groups were calculated as 6.012, 3.338, and 5.553, respectively. However, in Bonferroni-corrected pairwise comparisons, none of the differences between the control and addict groups (*p* = 0.067), the control and treatment groups (*p* = 1.000), or the addict and treatment groups (*p* = 0.093) reached statistical significance.

When age and BMI were controlled for together for miR-134-5p, the overall group effect was not found to be statistically significant (F(2,34) = 3.066; *p* = 0.060; partial η^2^ = 0.153). The independent effects of age (F(1,34) = 1.233; *p* = 0.275; partial η^2^ = 0.035) and BMI (F(1,34) = 0.072; *p* = 0.790; partial η^2^ = 0.002) were also not found to be significant. The adjusted means for the control, addict, and treatment groups were calculated as 8.807, 7.374, and 9.704, respectively. In Bonferroni-corrected pairwise comparisons, none of the differences between the control and addict groups (*p* = 0.584), the control and treatment groups (*p* = 1.000), or the addict and treatment groups (*p* = 0.060) reached statistical significance.

### 2.5. ROC Evaluation of miRNAs

**Figure 2:**
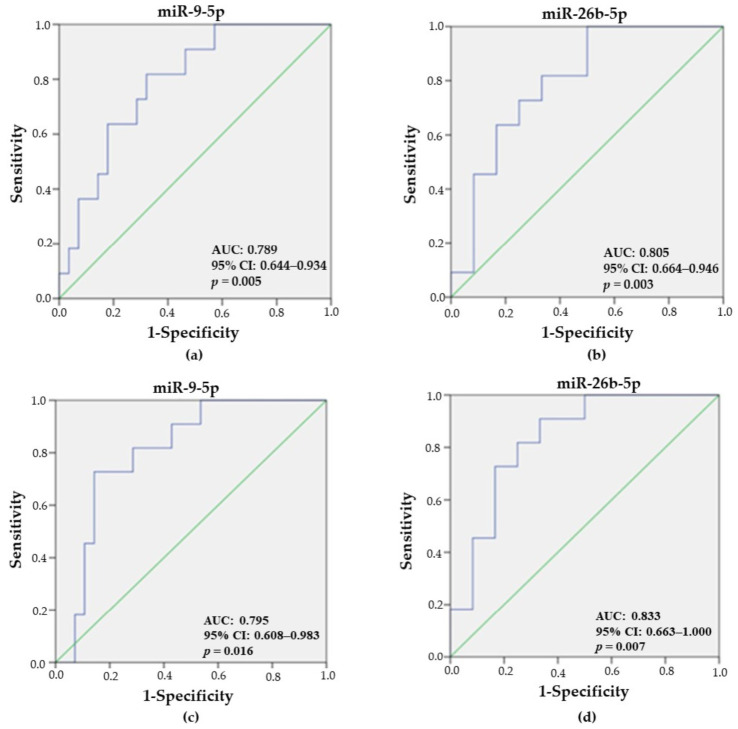

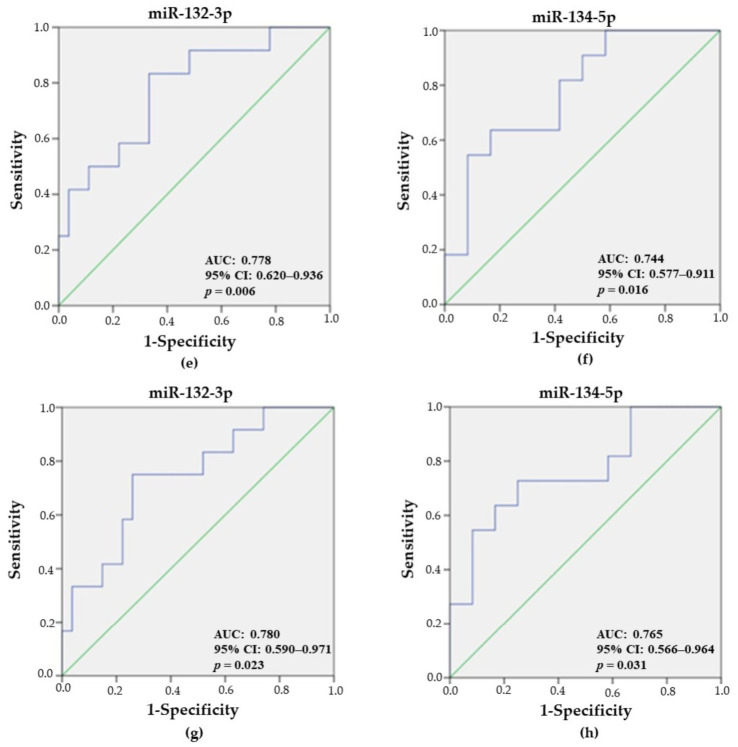
ROC curves illustrating the discriminatory performance of the evaluated miRNAs between groups. (**a**,**b**) show the discriminatory performance of miR-9-5p and miR-26-5p, respectively, between the control group and the treatment group. (**c**,**d**) show the discriminatory performance of miR-9-5p and miR-26-5p, respectively, between the addict group and the treatment group. (**e**,**f**) show the discriminatory performance of miR-132-3p and miR-134-5p, respectively, between the control group and the addict group. (**g**,**h**), in turn, show the discriminatory performance of miR-132-3p and miR-134-5p, respectively, between the addict group and the treatment group. The AUC value indicates the discriminatory power of the relevant miRNA between the two groups being compared. The blue line represents the ROC curve for the corresponding miRNA, while the green diagonal line represents the reference line indicating random classification performance (AUC = 0.5).

An ROC curve analysis was performed to assess the diagnostic potential of target miRNAs in distinguishing cocaine dependence and the discriminatory accuracy of miRNA expression data; the results are presented in Table 3. In the ROC curve analysis aimed at distinguishing the control group from the treatment group, the AUC values for miR-9-5p and miR-26-5p were calculated as 0.789 and 0.805, respectively; both values were found to be statistically significant (Figure 2a,b). In the ROC curve analysis aimed at distinguishing the addict group from the treatment group, the AUC values for miR-9-5p and miR-26-5p were calculated as 0.795 and 0.833, respectively; both values were found to be statistically significant (Figure 2c,d). In the ROC curve analysis aimed at distinguishing the control group from the addict group, the AUC values for miR-132-3p and miR-134-5p were calculated as 0.778 and 0.744, respectively; both values were found to be statistically significant (Figure 2e,f). In the ROC curve analysis aimed at distinguishing the addict group from the treatment group, the AUC values for miR-132-3p and miR-134-5p were calculated as 0.780 and 0.765, respectively; both values were found to be statistically significant (Figure 2g,h).

## 3. Discussion

In this study, the expression levels of target miRNAs in peripheral blood were evaluated among men with CUD, the treatment group, and the healthy control group. The findings revealed that some target miRNAs may exhibit different expression patterns across the groups. Although human studies examining changes in circulating miRNA expression in cocaine use disorder exist, peripheral blood data obtained specifically from men are limited. To the best of our knowledge, this study is the first to evaluate the expression of candidate miRNAs in peripheral blood in men with CUD.

Epigenetic profiles may be influenced by factors such as age, race, ethnicity, and an individual’s nutritional status, as well as by sex, which can contribute to interindividual differences [29]. Since substance dependence varies according to factors such as age, gender, and race, and since gender differences are one of the most significant factors influencing the onset and continuation of substance use, studies in the literature emphasize the need to match sample groups by gender, match them by age, stratify them by age, or adjust for age [30]. It is evident that there are limited studies in the literature examining the differences between women and men in the development of cocaine use disorder at the molecular level. It is evident from the literature that there are limited studies examining the differences between women and men in the development of cocaine use disorder at the molecular level. However, evaluating a homogeneous sample consisting solely of men is important for limiting the potential confounding effect of sex on miRNA expression levels. Furthermore, the fact that the proportion of male cocaine users worldwide is higher than that of females, that the proportion of males receiving addiction treatment is higher than that of females, and that the proportion of males among substance-related deaths is higher than that of females are additional reasons for including only male participants in this study. Furthermore, it has been noted that BMI, a marker of metabolic status, may also be associated with circulating miRNA levels [30]. In our current study, the fact that the mean age of the control group was lower than that of the other groups and that there were significant differences in BMI among the addiction, treatment, and control groups suggests that these variables may have potential confounding effects on the miRNA expression results. No statistically significant relationship was found between the ΔCt values of miRNAs assessed by BMI; furthermore, both variables were included as covariates in the analysis model to jointly evaluate the potential effects of BMI. When age and BMI were controlled for together, the overall group effect for miR-9-5p and miR-26b-5p was found to remain statistically significant. For both miRNAs, the adjusted ΔCt values in the treatment group were found to be significantly higher than those in the control and addict groups, while no significant difference was detected between the control and addict groups. These results indicate that the differences observed in the treatment group regarding miR-9-5p and miR-26b-5p cannot be explained solely by differences in age and BMI between the groups. Although the overall group effect for miR-132-3p, adjusted for age and BMI, was found to be statistically significant, none of the Bonferroni-corrected pairwise comparisons reached statistical significance. While these data indicate that a general difference between groups can be detected, they also show that it cannot be definitively determined in the current sample which groups account for this difference. Regarding miR-134-5p, when age and BMI were controlled for together, neither the overall group effect nor the Bonferroni-corrected pairwise comparisons were found to be statistically significant. The fact that the comparison between the addict and treatment groups was close to the threshold of statistical significance may indicate a numerical difference. However, this finding should not be interpreted as a statistically significant group difference. Furthermore, the independent effects of age and BMI were not found to be statistically significant for any of the miRNAs examined. Consequently, the validity of these findings must be examined through studies conducted with more comprehensive and independent sample groups.

Although BMI findings were evaluated in our current study in terms of the participants’ general physiological characteristics, the biological effects of cocaine addiction are not limited to anthropometric variables alone. Therefore, the effects of the addiction process on dopaminergic and glutamatergic signaling in brain regions associated with the reward system must also be taken into account. The course of CUD is associated with persistent neuroadaptive changes resulting from the continuous activation of dopaminergic and glutamatergic signaling in brain regions linked to the reward system. Recurrent cocaine exposure may contribute to the maintenance of drug-seeking behavior by modulating intracellular signaling pathways associated with dopamine D1 and NMDA receptors, ERK/CREB-mediated gene expression, dendritic architecture, and synaptic plasticity [31,32]. miRNAs may play a role in the development of cocaine-related neuroadaptation, learning, memory, and neuroinflammatory reactions by regulating the expression of target mRNAs involved in these processes at the post-transcriptional level [13,14]. The cellular composition and miRNA expression profiles of peripheral blood and brain tissue differ. Therefore, the findings obtained from blood and brain tissue in this study were not directly compared. In studies in the literature, miRNA changes reported in brain tissue have been interpreted not as findings that confirm or contradict the peripheral blood results in our current study, but as complementary findings that elucidate the potential biological roles of target miRNAs associated with cocaine exposure and addiction.

In our current study, peripheral blood miR-9-5p levels did not show a statistically significant difference between the addict group and the control group. In contrast, we observed a statistically significant increase in miR-9-5p levels in the treatment group compared to both the control group and the addict group. Gu et al. (2020) found that miR-9-3p expression levels in serum samples from methamphetamine-dependent individuals were significantly higher than those in control subjects [18]. Cabana-Domínguez et al. (2018) found that miR-9-5p expression levels in human dopaminergic neuron-like cells, measured 6 h after acute exposure to 5 μM cocaine for 30 min, were reduced compared to the control group [14]. These findings suggest that miR-9-5p may play a role in cocaine-regulated gene expression and cellular processes associated with addiction. Furthermore, since this study was based on an acute cell culture model, the results cannot be directly extrapolated to chronic cocaine use disorder or peripheral blood. In a different neuropsychiatric disease model, it has been shown that exosomal miR-9-5p released from neurons regulates the JAK/STAT3 signaling pathway by inhibiting SOCS2, thereby promoting the shift of microglial cells toward the pro-inflammatory M1 phenotype and increasing the production of pro-inflammatory cytokines [33]. Therefore, miR-9-5p has been associated with a pro-inflammatory effect in this model; however, it has not been established that the same mechanism applies in cocaine addiction. In our current study, the change in miR-9-5p detected in peripheral blood may be linked to cocaine-associated cellular and neuroinflammatory changes. Furthermore, since the SOCS2 and JAK/STAT3 signaling pathways were not directly examined in this study, a definitive assessment of miR-9-5p’s protective or pro-inflammatory role cannot be made. Furthermore, the changes in miR-9-5p expression observed in various biological samples in relation to cocaine exposure suggest that this miRNA may play a role in addiction-related molecular processes. Additionally, studies evaluating both peripheral and central nervous system samples are needed to shed further light on this relationship.

In our current study, we did not observe a statistically significant difference in miR-26-5p levels in peripheral blood between the addict group and the control group. However, we observed a statistically significant increase in miR-26-5p levels in the treatment group compared to both the control group and the addict group. Chen et al. (2013) reported that miR-26b expression in the hippocampus of rats with a cocaine-conditioned place preference increased during the acquisition of cocaine preference and that this change persisted during the extinction phase [13]. Furthermore, since the specific miRNA strand was not specified in that study, it cannot be definitively concluded that these data pertain directly to miR-26b-5p. In a recent model of nerve damage, it was demonstrated that miR-26b-5p reduces inflammation, reactive oxygen species production, and cellular damage by targeting the KDM6A/NOX4 axis [34]. While these findings suggest that miR-26b-5p may have protective and anti-inflammatory effects in certain neurological conditions, the change in miR-26b-5p expression observed in peripheral blood in our current study may be related to regulatory processes associated with inflammation and oxidative stress. Furthermore, since the KDM6A/NOX4 axis was not directly examined in this study and this mechanism has not been validated in cocaine use disorder, a definitive assessment based on a specific mechanism cannot be made. Furthermore, the fact that the observed changes in miR-26b-5p expression across studies were consistent in direction suggests that miR-26b may play a role in molecular processes associated with cocaine exposure. However, given that peripheral blood and brain tissue possess different biological characteristics, these findings should not be interpreted as directly equivalent results.

In our current study, we observed a statistically significant decrease in peripheral miR-132-3p levels in the addict group compared to the control group. While no significant difference was observed between the treatment group and the control group, we detected a statistically significant increase in miR-132-3p levels in the treatment group compared to the addict group. Experimental studies in the literature related to cocaine have shown that miR-132-3p expression can vary depending on cocaine exposure, the stage of addiction, and the brain region being evaluated [12,35,36]. For example, Sadakierska-Chudy et al. (2017) reported that in the striatum of rats self-administering cocaine, the increase in miR-132 and miR-212 expression persisted in the absence of cocaine exposure, and that miR-212 expression continued to rise even during a 10-day withdrawal period [12]. In addiction-prone rats, however, increased miR-132 expression was observed in the nucleus accumbens shell following extinction and cocaine-seeking tests [35]. In contrast, in a different model of cocaine self-administration, it was reported that miR-132 and miR-212 expression were suppressed in the caudate putamen; this finding demonstrated that the miRNA response may vary depending on the brain region and experimental conditions [36]. In the proposed mechanistic model explaining the regulation of miR-132/212 expression following cocaine self-administration, it has been suggested that dopamine D1 receptor-mediated cAMP/PKA signaling and NMDA receptor-mediated Ca^2+^/CaMK signaling may converge on CREB, and that miR-132/212 transcription can be regulated via the CREB–CBP/p300 –CRTC system [12]. In neuronal models, it has been shown that miR-132 plays a role in neuronal morphogenesis, dendritic growth, and activity-dependent synaptic plasticity by suppressing p250GAP expression [37,38]. On the other hand, a direct functional effect reducing cocaine consumption during prolonged cocaine exposure has been demonstrated not for miR-132, but for miR-212, which is in the same cluster [11]. Accordingly, existing research on cocaine addiction is not sufficient on its own to classify miR-132 as either a direct protective or pro-inflammatory miRNA. The findings suggest that miR-132 modulates cocaine-related neuroadaptations and synaptic plasticity depending on the brain region and stage of addiction [11,12,35,36]. In this context, the change in miR-132-3p expression detected in peripheral blood in our current study is likely associated with cocaine-related neuroadaptation and synaptic plasticity processes. Furthermore, since p250GAP and related signaling pathways were not directly examined in this study, no definitive conclusions regarding the mechanism can be drawn. Furthermore, both in this study and in previous research on miR-132, the differing expression levels of this miRNA observed in peripheral blood and brain tissue suggest that miR-132-3p may be regulated in a tissue-specific manner.

In our current study, we also observed a statistically significant decrease in peripheral miR-134-5p levels in the addict group compared to the control group. While no significant difference was observed between the treatment group and the control group, we detected a statistically significant increase in miR-134-5p levels in the treatment group compared to the addict group. For example, it has been reported that miR-134 expression in the hippocampus of rats trained in cocaine-conditioned place preference exhibits changes during the acquisition and extinction of cocaine preference [13]. In Li et al., 2020, using a mouse model of cocaine-induced conditioned place preference [20], an increase in miR-134 levels associated with synaptic plasticity, glial activity, and neurochemical microenvironments was detected in the ventral hippocampus; it was also found that the absence of cocaine, when combined with a decrease in gene levels, increased anxiety- and depression-like behaviors in mice. Suppression of miR-134 in the ventral hippocampus partially reversed changes in dendritic spines and synaptic proteins. Furthermore, it reduced microglial activity, the inflammatory response, apoptosis, and markers of oxidative stress [20]. These findings suggest that increased miR-134-5p expression may be associated with adverse neuroplastic and pro-inflammatory processes in the relevant cocaine extinction mechanism. However, it is important to note that this conclusion should not be generalized to all tissues and disease conditions. Studies on the underlying mechanism have shown that miR-134 regulates dendritic spine size and synaptic architecture by suppressing the translation of LIMK1 mRNA, which plays a role in dendritic spine development [39]. Furthermore, it has been reported that SIRT1 and YY1 suppress miR-134 expression; increased miR-134 levels may negatively affect synaptic plasticity and memory processes by reducing CREB and BDNF expression [40]. In this context, the change in miR-134-5p expression detected in peripheral blood in our current study is likely associated with processes related to synaptic plasticity, microglial activation, inflammation, and oxidative stress. However, since the LIMK1 and SIRT1–YY1–miR-134–CREB/BDNF regulatory axes were not directly investigated in this study, these mechanisms should be considered as potential biological mechanisms underlying the findings in peripheral blood. In addition, both this study and previous research on miR-134-5p have shown that the expression of this miRNA differs between peripheral blood and brain tissue, suggesting that this miRNA may be regulated in a tissue-specific manner.

In our study, comparisons of miRNA expression between the patient, treatment, and control groups revealed that the groups exhibited distinct expression profiles. Consequently, these observed changes in expression suggest that the analyzed miRNAs may be associated with the treatment process and could be investigated as potential biomarkers for assessing treatment response. At this point, the partial overlap in the study groups is noteworthy in terms of explaining potential miRNA changes associated with the treatment process. The addict and treatment groups partially overlapped for two patients, from whom blood samples were collected both before and after treatment. This situation made it possible to observe individual miRNA expression changes, even though the number of cases was limited. However, these observations, which are based on only two patients, are exploratory in nature and are not sufficient to draw definitive conclusions regarding treatment-related changes. The other individuals in the treatment group were already undergoing treatment at the clinic prior to the start of this study and were included in the study using only their post-treatment samples. Therefore, it is not possible to interpret the differences identified in the treatment group as changes directly attributable to treatment.

Furthermore, the type and duration of treatment administered at the clinic, as well as the time elapsed between the last cocaine use and blood sample collection, were not standardized across patients. No statistically significant relationship was found between the time since last use, the duration of detoxification, and the miRNA ΔCt values. Furthermore, this result did not completely rule out the possible effects of these clinical variables on miRNA expression. Therefore, the validity of the findings must be assessed through larger longitudinal studies in which treatment and sampling times are standardized and the same individuals are followed before and after treatment. To investigate whether peripheral blood miRNAs are associated with the clinical characteristics of cocaine-dependent individuals and patients undergoing addiction treatment, the correlations between the expression of miR-9-5p, miR-26b-5p, miR-132-3p, and miR-134-5p—as defined in the study—and substance use histories were analyzed. No statistically significant correlation was found between the expression of these four candidate miRNAs in the addiction and treatment groups and characteristics such as duration of substance use, daily dose, type of cocaine used, frequency of use, and time since last use (*p* > 0.05). These findings suggest that changes in miRNA expression levels may reflect not only biological differences between groups but also potential discriminatory features. Therefore, the ability of the relevant miRNAs to distinguish between groups was evaluated using ROC analysis. In this study, we performed ROC curve analyses to evaluate the diagnostic validity of miR-9-5p, miR-26b-5p, miR-132-3p, and miR-134-5p for CUD, both between the treatment group and the control group and between the treatment group and the group of individuals with CUD. The significant results obtained from the ROC analyses provide preliminary evidence that the four candidate miRNAs analyzed may play a potential role in distinguishing between the substance-addict, treatment, and control groups. Furthermore, it is considered necessary to evaluate the feasibility of using these four miRNAs as diagnostic biomarkers in larger samples and independent validation cohorts.

The results of this study should be interpreted in light of certain limitations. First, although a sufficient number of individuals were initially included in the addict, treatment, and control groups, some participants were excluded from the groups prior to the analysis phase and were not included in the study due to low RNA concentrations in their peripheral blood. This resulted in a reduction in the number of samples in each group. Therefore, due to the small sample size, the experiments were repeated twice to ensure statistical significance. Additionally, the small sample size may have led to an insufficient analysis of the relationship between miRNA and a history of cocaine use. Second, we did not perform mRNA or protein analysis regarding the predicted targets of the miRNAs. Furthermore, while our study consisted solely of male participants—which helped limit gender-related biological variability—it also restricts the generalizability of the findings to women.

Despite these limitations, the results of this study demonstrate that the expression of miR-132-3p and miR-134-5p in peripheral blood differs significantly between male subjects with CUD and healthy male controls. Together with the evidence accumulated to date, the findings of our study suggest that the candidate miRNAs investigated have the potential to reflect molecular changes associated with cocaine use disorder and treatment status [41,42,43]. Furthermore, the data obtained should be interpreted as preliminary and exploratory. In future prospective studies conducted with more comprehensive and independent sample groups, it will be important to monitor pre- and post-treatment periods in the same individuals, standardize treatment and sampling protocols, and interpret miRNA expression changes in conjunction with clinical treatment response. Furthermore, investigating the relevant target genes and signaling pathways using functional methods and testing the results obtained from ROC analyses in independent validation cohorts will help determine the biological and clinical relevance of these miRNAs using more reliable methods. The results obtained in this study may serve as a foundation for evaluating candidate biomarkers that could reflect the molecular changes occurring during cocaine use disorder and the withdrawal process in future research.

## 4. Materials and Methods

### 4.1. Participants

Based on self-report and urine screening of participants selected from Istanbul Balikli Greek Hospital Anatolia Psychiatry Clinic, a total of 12 male cocaine addict individuals (*n* = 12) who were addicted to cocaine and had just presented for treatment for the addict group and 11 male cocaine addict individuals (*n* = 11) who were addicted to cocaine and had just presented for treatment and/or were currently undergoing treatment for cocaine dependence at the clinic for the treatment group were included in this study. For the healthy control group, 16 male volunteers (*n* = 16) were recruited who had never used cocaine, had no substance dependence, and were not related to each other.

The addict group consisted of patients enrolled in the study before receiving any addiction treatment. The treatment group, on the other hand, consisted of two subgroups. The first subgroup included two patients with addiction who were initially enrolled in the addict group, subsequently received addiction treatment, and had blood samples collected again after treatment. Blood samples were collected from these patients at two different time points—before and after treatment—and the patients were evaluated in both groups. The second subgroup consisted of patients who had received and completed addiction treatment at the clinic before the study began and were included in the study solely based on blood samples collected after treatment. Therefore, the addict and treatment groups partially overlapped with regard to two participants.

In the Treatment Center of the clinic, patients diagnosed with CUD according to DSM-5 diagnostic criteria receive detoxification treatment and/or Vanoxerine treatment according to their comorbidity. The treatment approach and duration applied to individuals in the treatment group were determined based on the patients’ clinical characteristics, comorbidities, and individual treatment needs. Consequently, the type and duration of treatment were not standardized across participants. In general, detoxification treatment was administered to patients in accordance with their clinical needs, and the duration of detoxification was recorded in days. The time elapsed between the last cocaine use and blood sample collection was not standardized, as it varied among individuals in both the addiction and treatment groups. Additionally, for individuals in both groups, the time elapsed since the last cocaine use was recorded in days.

All participants included in the study identified themselves as being of Turkish ethnic origin. Written informed consent was obtained from all participants prior to enrollment. The molecular genetic analysis of the samples taken from the participants was carried out at the Institute’s Forensic Molecular Genetics Laboratory. The inclusion and exclusion criteria for the addict and treatment groups in this study are as follows: (a) being between the ages of 18 and 65, (b) Being diagnosed with CUD according to DSM-V diagnostic criteria (ICD-10 F14), and (c) no major psychiatric disorder, or substance abuse other than cocaine (as determined by urine drug testing and/or self-report of the participant). The inclusion criteria for the healthy control group were as follows: (a) age between 18 and 65 years, (b) no drug use, (c) not having any substance addiction, and (d) not diagnosed with any medical or psychiatric disorder (as determined by the participant’s declaration). Pregnant women and children under 18 years of age were not included in the study and intellectual disability was determined as an exclusion criterion for all 3 groups.

### 4.2. Study Procedures

Patients were assessed for psychiatric diagnosis and history of substance abuse during the first week of treatment. Body mass index (BMI) data were assessed for each participant. Blood samples were collected only once from all participants. However, some volunteer patients in the addict group also had blood samples collected after their treatment.

### 4.3. Selection of Candidate miRNAs for qPCR Analysis

In this study, candidate miRNAs thought to play a role in the regulation of cocaine addiction were identified using the search terms “addiction,” “substance abuse,” “cocaine addiction,” “cocaine use disorder,” “microRNA,” “addiction,” “substance abuse,” “cocaine addiction,” “cocaine use disorder,” and “microRNA.” Additionally, these candidate miRNAs were identified by screening the “miRBase” (https://ngdc.cncb.ac.cn/databasecommons/database/id/165, accessed on 5 July 2026) and “TargetScan” (http://www.targetscan.org/) databases and analyzing the collected data. In this study, two housekeeping (reference) genes, SNORD44 and SNORD48, were selected as normalizers. miR-9-5p, miR-26b-5p, miR-132-3p, and miR-134-5p were selected as candidate miRNAs.

In this study, the candidate miRNAs evaluated in the qPCR analysis were selected based on previous reports indicating that they exhibited altered expression in brain tissue samples associated with cocaine exposure, and on recommendations to investigate these miRNAs in various biological samples. However, a review of the current literature revealed that the expression levels of these miRNAs in the peripheral blood of individuals with cocaine use disorder had not previously been evaluated. Therefore, the present study focused on peripheral blood expression levels to investigate whether the findings reported in brain tissue are reflected in peripheral blood and to evaluate the potential biomarker properties of these miRNAs in an easily accessible biological sample.

### 4.4. Analysis of Peripheral Blood miRNA Levels

In this study, total RNA was isolated from peripheral blood. PAXgene Blood miRNA Kit (Cat. No. 763134; Qiagen, Hilden, Germany) was used for total RNA isolation. Since blood samples were stored at −70 °C after collection, after equilibration to room temperature, total RNA was extracted following the steps in the kit protocol and reconstituted in 40 μL RNase-free water. The amount of RNA was measured with the RNA HS (High Sensitivity) Assay Kit (Cat. No. Q32852; Invitrogen, Carlsbad, CA, USA) on a Qubit^®^ Fluorometer (Invitrogen, Carlsbad, CA, USA). The total RNA obtained was stored at −70 °C until further experimental studies. More stable cDNA was obtained from the isolated total RNAs by reverse transcription (RT) with miRCURY LNA RT Kit (Cat. No. 339340; Qiagen, Hilden, Germany). RT-qPCR was performed on a Rotor-Gene Q instrument (Qiagen, Hilden, Germany) using the miRCURY LNA SYBR Green PCR Kit (Cat. No. 339346; Qiagen, Hilden, Germany). The following Qiagen primers were used: Hs miR-9-5p (Cat. No. 339306-YP00204513; Qiagen, Hilden, Germany), Hs miR-26b-5p (Cat. No. 339306-YP00204172; Qiagen, Hilden, Germany), Hs miR-132-3p (Cat. No. 339306-YP00206035; Qiagen, Hilden, Germany), and Hs miR-134-5p Hs miR-134-5p (Cat. No. 339306-YP00205989; Qiagen, Hilden, Germany). Each sample was amplified twice in qRT-PCR. SNORD44 (Cat. No. 339306-YP00203902; Qiagen, Hilden, Germany) and SNORD48 (Cat. No. 339306-YP00203903; Qiagen, Hilden, Germany) were used as reference genes to normalize target miRNAs to calculate ΔCt values. The ΔCt method was used for relative expression evaluation.

### 4.5. Statistical Analysis

Statistical analysis was performed using IBM SPSS Statistics 20. One-way ANOVA test was used to analyze whether there was a difference in ΔCt values of candidate miRNAs between addict, treatment and control groups. LSD test or Tamhane’s T2 test was used as post hoc test.

The correlation between the expression of miRNAs and the clinical data of the addict, treatment and control group individuals was determined using Pearson correlation analysis in normally distributed groups and Spearman correlation test in non-normally distributed groups. To control for the potential confounding effects of age and BMI differences between the addict, treatment, and control groups, an ANCOVA was performed for each target miRNA, with age and BMI included as covariates. Homogeneity of regression slopes was assessed using group × age and group × BMI interactions. Additionally, homogeneity of error variances was examined using the Levene test. In adjusted pairwise group comparisons, the Bonferroni correction was applied, and effect sizes were expressed as partial eta-squared (η^2^). ROC analysis method was used to analyze the diagnostic performance of the variables. With ROC analysis, appropriate estimation points, Sensitivity, Specificity values and AUCs were examined for the variables. In all analyses, *p* < 0.05 was considered statistically significant.

## Figures and Tables

**Table 1:** Sociodemographic and clinical characteristics of the sample.

Variable	Controls (*n* = 16)	Addict Group (*n* = 12)	*p*-Value ^a^	Treatment Group (*n* = 11)	*p*-Value ^b^	*p*-Value ^c^
**Age (years), mean ± SD**	22.25 ± 5.94	35.17 ± 5.86	<0.001	36.55 ± 10.76	0.004 ^d^	0.976 ^e^
**BMI (kg/m^2^), mean ± SD**	22.79 ± 2.38	26.57 ± 4.32	0.006	25.46 ± 3.40	0.050 ^f^	0.436 ^g^
**Drug use history**						
**Substance use preference, *n* (%)**						
**Cocaine**	-	12 (100)	-	12 (100)	-	-
**Alcohol**	-	8 (66.7)	-	7 (63.6)	-	-
**Cigarette**	-	12 (100)	-	10 (90.9)	-	-
**Cannabis**	-	3 (25)	-	3 (27.3)	-	-
**Duration of use (months), *n* (%)**						
**<12**	-	4 (33.3)	-	2 (18.2)	-	-
**12–24**	-	1 (8.3)	-	4 (36.4)	-	-
**24–48**	-	5 (41.7)	-	2 (18.2)	-	-
**48–96**	-	2 (16.7)	-	1 (9.1)	-	-
**≥96**	-	-	-	2 (18.2)	-	-
**Daily dose (g), *n* (%)**						
**<1**	-	1 (8.3)	-	1 (9.1)	-	-
**1–2**	-	5 (41.7)	-	5 (45.5)	-	-
**>2**	-	6 (50)	-	5 (45.5)	-	-
**Type of cocaine used, *n* (%)**						
**Powder cocaine**	-	8 (66.7)	-	10 (90.9)	-	-
**Crack cocaine**	-	4 (33.3)	-	1 (9.1)	-	-
**Frequency of use, *n* (%)**						
**<1 times/d**	-	3 (25)	-	3 (27.3)	-	-
**1–3 times/d**	-	9 (75)	-	8 (72.7)	-	-
**Most recent use (days), *n* (%)**						
**<1**	-	6 (50)	-	5 (45.5)	-	-
**1–3**	-	4 (33.3)	-	-	-	-
**3–7**	-	1 (8.3)	-	2 (18.2)	-	-
**>7**	-	1 (8.3)	-	-	-	-
**>10**	-	-	-	4 (36.4)	-	-
**Frequency of detoxification, *n* (%)**						
**<2**	-	10 (83.3)	-	7 (63.6)	-	-
**2–4**	-	1 (3.3)	-	4 (36.4)	-	-
**>4**	-	1 (3.3)	-	-	-	-
**Treatment type, *n* (%)**						
**Detoxification**	-	-	-	10 (90.9)	-	-
**Vanoxerin**	-	-	-	1 (9.1)	-	-

BMI: Body Mass Index. Continuous variables are presented as mean ± standard deviation, while categorical variables are presented as *n* (%). *n* represents the number of participants in the respective category; the values in parentheses indicate the percentages within the respective group. ^a^: Comparison of the addict group with the control group; ^b^: Comparison of the treatment group with the control group; ^c^: Comparison of the addict group with the treatment group; ^d^ and ^e^: Kruskal–Wallis test; ^f^ and ^g^: One-way analysis of variance (ANOVA).

**Table 2:** Comparison of ΔCt values between groups with relative miRNA expression levels in peripheral blood.

	Groups ΔCt ^a^ (Mean ± SD)			Compared Groups	
miRNA	Addict (*n* = 12)	Treatment (*n* = 11)	Control (*n* = 16)	*p*-Value ^b^	
miR-9-5p	10.73 ± 2.18	12.74 ± 1.29	10.98 ± 1.84		
				Treatment-Control	0.019 ^b^
				Treatment-Addict	0.012 ^b^
				Addict-Control	0.723
miR-26b-5p	0.10 ± 2.43	3.01 ± 1.77	0.68 ± 2.99		
				Treatment-Control	0.024 ^b^
				Treatment-Addict	0.009 ^b^
				Addict-Control	0.549
miR-132-3p	3.32 ± 2.61	5.50 ± 1.47	6.06 ± 2.42		
				Addict-Control	0.003 ^b^
				Treatment-Addict	0.026 ^b^
				Treatment-Control	0.534
miR-134-5p	7.14 ± 2.57	9.36 ± 1.63	9.21 ± 2.31		
				Addict-Control	0.020 ^b^
				Treatment-Addict	0.023 ^b^
				Treatment-Control	0.863

^a^ Relative miRNA expression levels in peripheral blood are presented as ΔCt values. ΔCt values are dimensionless and are expressed as mean ± standard deviation (SD). ^b^ One-way ANOVA.

**Table 3:** Results of ROC analysis to assess the diagnostic adequacy of peripheral blood miRNAs.

Compared Groups	Groups	miRNAs	Cut Off	Sensitivity	Specificity	AUC	% 95 CI	*p*-Value
Control– Treatment	Treatment	miR-9-5p	12.3450	72.7	71.4	0.789	0.644–0.934	0.005
	Treatment	miR-26b-5p	1.9100	72.7	71.4	0.805	0.664–0.946	0.003
Addict– Treatment	Treatment	miR-9-5p	12.3450	72.7	75	0.795	0.608–0.983	0.016
	Treatment	miR-26b-5p	1.9100	72.7	75	0.833	0.663–1.000	0.007
Control–Addict	Addict	miR-132-3p	5.4038	88.3	66.7	0.778	0.620–0.936	0.006
	Addict	miR-134-5p	8.3825	75	25.9	0.744	0.577–0.911	0.016
Addict– Treatment	Treatment	miR-132-3p	5.2388	63.6	66.7	0.780	0.590–0.971	0.023
	Treatment	miR-134-5p	8.8898	72.7	75	0.765	0.566–0.964	0.031

## Data Availability

The data supporting the findings of this study are not publicly available due to ethical and privacy restrictions, but are available from the corresponding author upon reasonable request.

## Abbreviations

The following abbreviations are used in this manuscript:

miRNA	MicroRNA
ncRNA	Non-coding RNA
RT-qPCR	Quantitative reverse-transcription
ROC	Receiver operating characteristic
AUC	Area under of the curve
Cq	Quantification cycle
CUD	Cocaine use disorder
BMI	Body mass index
